# Acetylation of p65 at lysine 314 is important for late NF-κB-dependent gene expression

**DOI:** 10.1186/1471-2164-11-22

**Published:** 2010-01-11

**Authors:** Karin M Rothgiesser, Monika Fey, Michael O Hottiger

**Affiliations:** 1 Institute of Veterinary Biochemistry and Molecular Biology, University of Zurich, Winterthurerstrasse 190, 8057 Zurich, Switzerland

## Abstract

**Background:**

NF-κB regulates the expression of a large number of target genes involved in the immune and inflammatory response, apoptosis, cell proliferation, differentiation and survival. We have earlier reported that p65, a subunit of NF-κB, is acetylated *in vitro *and *in vivo *at three different lysines (K310, K314 and K315) by the histone acetyltransferase p300.

**Results:**

In this study, we describe that site-specific mutation of p65 at lysines 314 and 315 enhances gene expression of a subset of NF-κB target genes including *Mmp10 *and *Mmp13*. Increased gene expression was mainly observed three hours after TNFα stimulation. Chromatin immunoprecipitation (ChIP) experiments with an antibody raised against acetylated lysine 314 revealed that chromatin-bound p65 is indeed acetylated at lysine 314.

**Conclusions:**

Together, our results establish acetylation of K314 as an important regulatory modification of p65 and subsequently of NF-κB-dependent gene expression.

## Background

The inducible transcription factor NF-κB has an important function in regulating immune and inflammatory responses, apoptosis, cell proliferation and differentiation and tumorigenesis [1-3]. NF-κB is induced in almost all cell types by different extracellular stimuli causing the activation of an enormous array of target genes [4]. The NF-κB transcription factor family comprises NFKB1 (p50/p105), NFKB2 (p52/p100), p65 (RelA), c-Rel and RelB, which form homo- and heterodimers. The most abundant, best-studied and "classical" form of NF-κB is a heterodimer consisting of the two subunits p50 and p65. In most unstimulated cells, NF-κB is found as inactive transcription factor complex through its physical association with one of the several inhibitors of NF-κB (IκB) [5]. This family of IκB's includes IκBα, IκBβ, IκBγ, IκBε (p105/p50, C-terminus), p100/p52 (C-terminus), IκB-R and Bcl-3. Virtually all cell types show NF-κB responses where the activity of NF-κB is specifically regulated at multiple levels [1,3,6]: the level of protein synthesis, the existence of at least 12 different NF-κB dimers, the interaction of these dimers with specific IκBs and their subcellular localization, post-translational modification of these dimers in the cytoplasm and the nucleus, differential accessibility of κB sites in various promoter and enhancer, differential binding to κB's response elements due to different affinities, and cell type and stimuli specific interaction with a combination of cofactors.

NF-κB is subject to a variety of post-translational modifications (e.g., phosphorylation [7], ubiquitination [8] or prolyl-isomerisation [9]) that modulate its activity. Phosphorylation of the p65 subunit by the PKAc, MSK1 and PKCζ kinases enhances its interaction with the co-activator p300/CBP and stimulates the NF-κB transcriptional activity [7,10-12], while dephosphorylation of p65 by the phosphatase WIP1 negatively affected the interaction with p300 [13]. It has recently been shown that p65 and p50 are reversibly acetylated by p300 and PCAF [14-16]. Chen *et al. *identified lysine residues (K) 218, 221 and 310 of p65 as acceptor sites for p300 acetylation. Kiernan *et al*. identified K122 and 123 in p65 as acetylation sites modified by both p300 and P/CAF. A recent report presented the TGF-β1-mediated acetylation of p65 at K221 *in vitro *and *in vivo *enhancing the induced activation of NF-κB by bacteria [17]. We recently confirmed acetylation on K310 and provided further evidence for acetylation of p65 on K314 and 315, two novel acetylation sites [18]. Genetic complementation of *p65 *knockout (-/-) cells with wild type and acetylation-deficient mutants of p65 revealed that neither shuttling, DNA binding nor the induction of anti-apoptotic genes by TNFα was affected by acetylation on these residues. Micro array analysis of these cells treated with TNFα for only 45 minutes identified specific sets of genes differently regulated by wild type or acetylation-deficient mutants of p65 [18]. Specific genes were either stimulated or repressed by the acetylation-deficient mutants when compared to p65 wild type. These results support the hypothesis that site-specific p300-mediated acetylation of p65 regulates the specificity of NF-κB dependent gene expression.

Here, we extended the gene expression analysis to three hours after TNFα stimulation and identified genes, which are higher expressed by mutating K314 and 315. ChIP experiments with antibodies directed against acetylated K314 revealed that this lysine is indeed acetylated when p65 is bound to chromatin. Together our results provide evidence that acetylation of K314 is important for the regulation of NF-κB-dependent gene expression *in vivo*.

## Results

### Mutation of p65 K314/315 regulates TNFα-induced NF-κB-dependent gene expression at 3 hours

**Figure 1:**
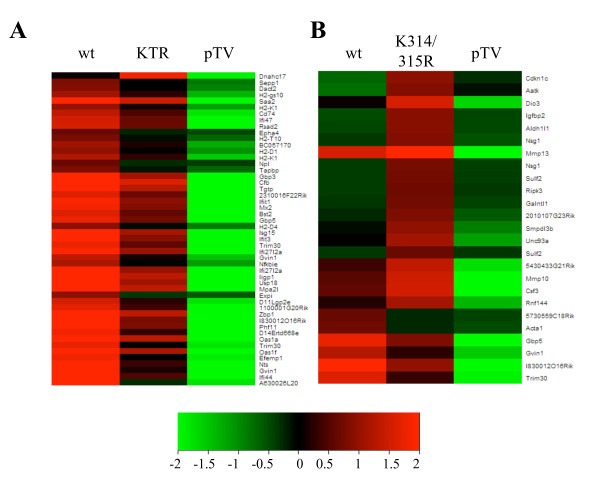
**Site-specific acetylation of p65 regulates the expression of distinct genes**. Heat maps showing the gene expression profiles of KTR cells **(A) **or K314/315R cells **(B) **compared to wild type control at 3 hours after TNFα stimulation using whole mouse genome arrays. Each row represents a single gene, and each column represents a different cell line. Red or green represents up- or downregulation of genes relative to the mean on each row, respectively. Mean data from at least two biological replicates is displayed. Only genes upregulated in wild type cells compared to pTV control were taken into account (>2-fold, p-value < 0.05). From these genes, only the ones with significant changes in expression levels (> 1.8-fold or < 0.556-fold, p-value < 0.05) between wild type and mutant cells are shown.

We provided earlier evidence that acetylation of p65 at K310, 314 and 315 is important for the expression of a defined subset of genes [18]. These earlier studies provided a first glance of the functional relevance of p65 acetylation, since gene expression was measured only after 45 minutes of TNFα stimulation. In order to know if the requirement for site-specific acetylation is maintained for the same genes after longer exposure to TNFα, and to identify possible new genes regulated through p65 acetylation, we decided to extend our analysis to 3 hours of stimulation. For this, we used *p65*(-/-) mouse embryonic fibroblasts (MEFs) complemented with acetylation-deficient mutants, where the target lysines for acetylation were mutated to arginines. These cells were described and extensively characterized previously [18]. *p65*(-/-) cells complemented either with wild type p65, an empty plasmid as control (pTV), the acetylation-deficient double mutant K314/315R or the triple mutant KTR (K310/314/315R), were stimulated by TNFα for 3 hours and total RNA was isolated in three independent replicates from these cells. RNA was amplified, labeled and hybridized to the Agilent Whole Mouse Genome Array. After statistical analysis of the expression profiles, differentially expressed genes were identified (Fig. 1A-B, and Tables 1 and 2). We focused only on genes that required p65 for their proper induction, which were identified by comparing gene expression profiles from wild type and pTV cells. The majority of differentially expressed genes in KTR mutant were strongly downregulated compared to wild type cells, suggesting that acetylation of p65 at these residues is also an important modification for the expression of the extended NF-κB-dependent gene expression (Fig. 1A). In contrast, experiments with the double K314/315R mutant revealed that the majority of genes were slightly upregulated after 3 hours compared to wild type cells (Fig. 1B).

**Table 1:** Down- and upregulated genes in KTR cell line compared to wild type control

RefSeq RNA	Gene symbol	Gene name	Fold change	P-value
XR_001627	A630026L20	Hypothetical protein A630026L20	0.188	9.40E-30

NM_029000	Gvin1	GTPase, very large interferon inducible 1	0.26	8.04E-09

NM_024435	Nts	Neurotensin	0.262	5.50E-10

NM_133871	Ifi44	Interferon-induced protein 44	0.282	0.001

NM_146015	Efemp1	Epidermal growth factor-containing fibulin-like extracellular matrix protein 1	0.286	2.31E-20

NM_145153	Oas1f	2'-5' oligoadenylate synthetase 1F	0.349	0.000045

NM_199015	D14Ertd668e	DNA segment, Chr 14, ERATO Doi 668, expressed	0.368	0.000005

NM_145211	Oas1a	2'-5' oligoadenylate synthetase 1A	0.369	0.001

NM_172603	Phf11	PHD finger protein 11	0.371	3.08E-07

NM_009099	Trim30	Tripartite motif protein 30	0.378	0.001

NM_183249	1100001G20Rik	RIKEN cDNA 1100001G20 gene	0.395	8.10E-07

NM_030150	Dhx58	DEXH (Asp-Glu-X-His) box polypeptide 58	0.413	0.000006

NM_021394	Zbp1	Z-DNA binding protein 1	0.419	0.000443

NM_008690	Nfkbie	Nuclear factor of kappa light polypeptide gene enhancer in B-cells inhibitor, epsilon	0.422	0.000005

XM_001000862	I830012O16Rik	RIKEN cDNA I830012O16 gene	0.422	0.002

NM_021792	Iigp1	Interferon inducible GTPase 1	0.427	0.000001

NM_194336	Mpa2l	Macrophage activation 2 like	0.435	0.039

NM_007969	Expi	Extracellular proteinase inhibitor	0.436	0.000025

NM_029000	Gvin1	GTPase, very large interferon inducible 1	0.438	0.000024

NM_009099	Trim30	Tripartite motif protein 30	0.454	0.000002

NM_011909	Usp18	Ubiquitin specific peptidase 18	0.458	0.002

NM_029803	Ifi27l2a	Interferon, alpha-inducible protein 27 like 2A	0.46	0.000163

NM_029803	Ifi27l2a	Interferon, alpha-inducible protein 27 like 2A	0.462	0.000471

NM_008200	H2-D4	Histocompatibility 2, D region locus 4	0.483	0.000006

NM_010501	Ifit3	Interferon-induced protein with tetratricopeptide repeats 3	0.488	0.000741

NM_153564	Gbp5	Guanylate binding protein 5	0.493	0.000843

NM_013606	Mx2	Myxovirus (influenza virus) resistance 2	0.509	0.000002

NM_198095	Bst2	Bone marrow stromal cell antigen 2	0.511	0.000046

NM_009318	Tapbp	TAP binding protein	0.511	0.004

NM_172777	BC057170	cDNA sequence BC057170	0.519	0.000028

NM_018734	Gbp3	Guanylate binding protein 3	0.52	0.000867

NM_001001892	H2-K1	Histocompatibility 2, K1, K region	0.522	7.26E-07

NM_011579	Tgtp	T-cell specific GTPase	0.522	0.000163

NM_008198	Cfb	Complement factor B	0.523	0.000011

NM_010395	H2-T10	Histocompatibility 2, D region locus 1	0.527	0.000001

NM_007936	Epha4	Eph receptor A4	0.528	0.001

NM_010545	Cd74	CD74 antigen (invariant polypeptide of major histocompatibility complex, class II antigen-associated)	0.532	2.94E-09

NM_008331	Ifit1	Interferon-induced protein with tetratricopeptide repeats 1	0.532	0.002

NM_001001892	H2-K1	Histocompatibility 2, K1, K region	0.533	5.43E-08

NM_028749	Npl	N-acetylneuraminate pyruvate lyase	0.533	0.000004

NM_010380	H2-D1	Histocompatibility 2, D region locus 1	0.533	0.000007

NM_015783	Isg15	ISG15 ubiquitin-like modifier	0.534	0.008

NM_173743	2310016F22Rik	RIKEN cDNA 2310016F22 gene	0.535	0.000166

NM_011314	Saa2	Serum amyloid A 2	0.536	3.53E-07

NM_008330	Ifi47	Interferon gamma inducible protein 47	0.542	0.000013

NM_172826	Dact2	Dapper homolog 2, antagonist of beta-catenin (xenopus)	0.544	0.008

NM_021384	Rsad2	Radical S-adenosyl methionine domain containing 2	0.547	0.005

NM_001143689	H2-gs10	MHC class I like protein GS10	0.55	0.000007

NM_009155	Sepp1	Selenoprotein P, plasma, 1	0.552	1.58E-14

XM_126677	Dnahc17	Dynein, axonemal, heavy chain 17	3.081	2.80E-45

List of down- and upregulated genes in KTR cell line compared to wild type control after 3 hours of TNFα treatment, as measured by whole genome arrays. Average values from at least two biological replicates are shown.

**Table 2:** Differentially regulated genes in K314/315R versus wild type control

RefSeq RNA	Gene symbol	Gene name	Fold change	P-value
NM_007377	Aatk	Apoptosis-associated tyrosine kinase	2.995	7.21E-29

NM_009876	Cdkn1c	Cyclin-dependent kinase inhibitor 1C (P57)	2.979	1.99E-09

NM_172119	Dio3	Deiodinase, iodothyronine type III	2.685	2.31E-07

NM_027406	Aldh1l1	Aldehyde dehydrogenase 1 family, member L1	2.39	1.35E-09

NM_008342	Igfbp2	Insulin-like growth factor binding protein 2	2.381	0.000196

NM_010942	Nsg1	Neuron specific gene family member 1	2.254	0.000001

NM_008607	Mmp13	Matrix metallopeptidase 13	2.222	4.57E-07

NM_010942	Nsg1	Neuron specific gene family member 1	2.119	0.000013

NM_028072	Sulf2	Sulfatase 2	2.1	0.000004

NM_027251	2010107G23Rik	RIKEN cDNA 2010107G23 gene	2.057	1.81E-07

NM_019955	Ripk3	Receptor-interacting serine-threonine kinase 3	2.047	5.51E-10

NM_133888	Smpdl3b	Sphingomyelin phosphodiesterase, acid-like 3B	2.04	8.17E-19

NM_001081421	Galntl1	UDP-N-acetyl-alpha-D-galactosamine:polypeptide N-acetylgalactosaminyltransferase-like 1	2.005	0.000414

NM_028072	Sulf2	Sulfatase 2	1.956	1.26E-07

NM_199252	Unc93a	Unc-93 homolog A (C. elegans)	1.938	0.000978

XM_283765	5430433G21Rik	RIKEN cDNA 5430433G21 gene	1.915	0.00025

NM_080563	Rnf144	Ring finger protein 144	1.83	0.006

NM_019471	Mmp10	Matrix metallopeptidase 10	1.829	0.000263

NM_009971	Csf3	Colony stimulating factor 3 (granulocyte)	1.807	0.000516

NM_029000	Gvin1	GTPase, very large interferon inducible 1	0.501	0.005

NM_009099	Trim30	Tripartite motif protein 30	0.506	0.036

AK077243	I830012O16Rik	RIKEN cDNA I830012O16 gene	0.509	0.008

NM_009606	Acta1	Actin, alpha 1, skeletal muscle	0.511	4.48E-14

NM_153564	Gbp5	Guanylate binding protein 5	0.521	0.000007

NM_028872	5730559C18Rik	RIKEN cDNA 5730559C18 gene	0.523	8.43E-07

List of differentially regulated genes in K314/315R versus wild type control at 3 hours TNFα stimulation analyzed by microarrays. Average values from at least two biological replicates are shown.

### Gene expression of *Mmp10 *and *Mmp13 *is enhanced when K314/315 are mutated

**Figure 2:**
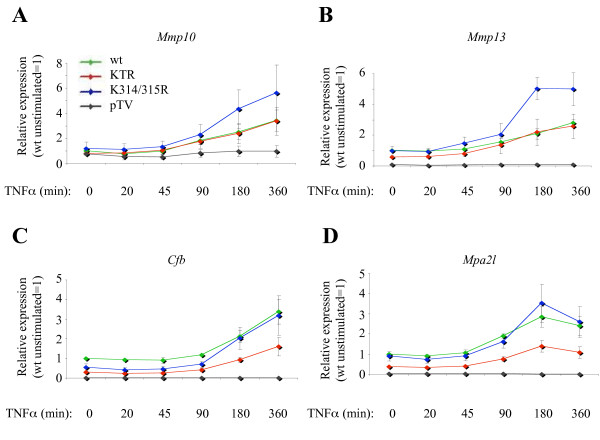
**Confirmation of gene expression profiles by qRT-PCR**. Gene induction of *Mmp10 *(A), *Mmp13 (B), Cfb (C) *and *Mpa2l ***(D) **in a TNFα-dependent manner, as measured by real-time RT-PCR, from the following complemented cell lines: wild type (green), KTR (red), K314/315R (blue) and pTV (grey). Samples were normalized to *Rps6 *and *CanX *expression levels, and expressed as fold increase relative to wild type unstimulated. Two biological replicates were included. Shown are the means ± SD of three independent runs.

We subsequently investigated the induction kinetics of several genes by real-time RT-PCR in the complemented cell lines stimulated by TNFα for different time points (between 20 and 360 minutes). Selection of these genes was based on their inducibility by TNFα, as well as their dependency on p65 and their regulation by acetylation of K314/315. *Mmp13 *and *Mmp10 *represented genes that were upregulated in the micro array experiments of K314/315R but not affected in KTR expressing cells. In contrast, *Cfb *and *Mpa2l *represented genes that were not affected in cells expressing K314/315R, but KTR, suggesting that acetylation of K310 is important for these genes. Overall, the absolute mRNA levels of *Mmp10 *and *Mmp13 *were strongly and significantly increased upon time (with a maximum value at 6 and 3 and hours, respectively) in cells expressing the K314/315R mutant (Fig. 2A, B and Additional file 1: Supplemental Table S1), while the expression levels of *Cfb *and *Mpa2l *were not affected in the same cells (Fig. 2C, D and Additional file 1: Supplemental Table S1), corroborating our micro array results. Gene expression analysis in cells expressing the KTR mutant indeed confirmed that K310 is important for *Cfb *and *Mpa2l*, but counteracts the effect of K314/315R for *Mmp10 *or *Mmp13 *expression. Interestingly, the dependency on K310 acetylation for *Cfb *and *Mpa2l *was already observed when the basal expression levels were analyzed (0 hour time point).

### Characterization of antibodies raised against acetylated lysine 314 and 315 of p65

**Figure 3:**
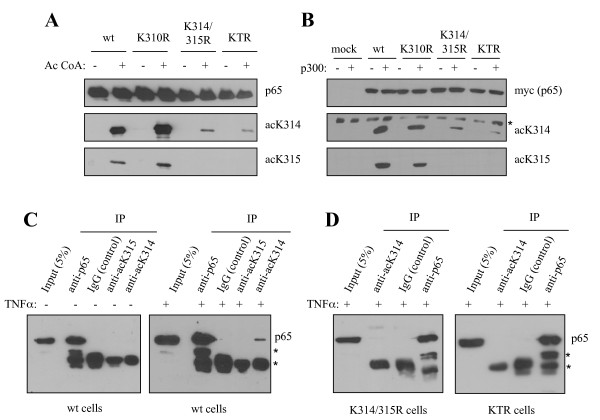
**Characterization of specific antibodies against p65 acetylated at K314 or 315**. **(A) **Purified recombinant p65 wild type and the acetylation-deficient mutants were incubated with recombinant p300 in the presence (+) or absence (-) of acetyl Co-A. Proteins were resolved on SDS-PAGE and analyzed by western blot using the indicated antibodies. **(B) **HEK 293T cells were transfected with p65 wild type or mutants, with (+) or without (-) p300 co-transfection. Acetylation of p65 at specific lysines was assessed by western blot using the specific antibodies. * indicates a non-specific band. (**C and D**) p65 was immunprecipitated from untreated or TNFα-stimulated cells using the indicated antibodies. Immunoprecipitated proteins were separated by 10% SDS-PAGE and subsequently analyzed by western blot using an anti-p65 antibody. * indicates IgG-band.

To further assess the functional relevance of p65 acetylation *in vivo*, we generated different antibodies raised against the acetylated K314 or K315. All raised antibodies recognized their specific p300-mediated acetylated residues on recombinant p65 acetylated *in vitro *(Fig. 3A) or on over expressed p65 acetylated *in vivo *(Fig. 3B).

To confirm that endogenous p65 is indeed acetylated at the indicated lysines, immunoprecipitation experiments with the antibodies raised against acetylated K314, K315 or against p65 were performed. The antibody anti-acK314 immunoprecipitated p65 only in cells complemented with wild type p65 in a TNFα-dependent manner (Fig. 3C). No p65 was immunoprecipitated in cells expressing the K314/315R or the KTR p65 mutant (Fig. 3D), suggesting, that endogenous p65 is indeed acetylated at K314 upon stimulation with TNFα. Unfortunately, anti-acK315 was not specific enough to recognize endogenous acetylation at K315.

### Chromatin-associated p65 is acetylated at lysine 314

**Figure 4:**
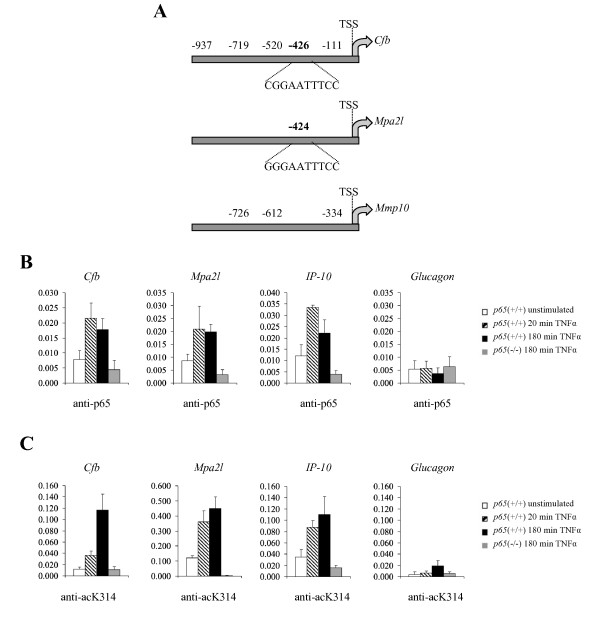
**Acetylated p65 is recruited to the promoter region of regulated genes upon TNFα stimulation**. **(A) **Promoters of *Cfb, Mpa2l *and *Mmp10 *have putative κB sites. Schematic representation of putative κB sites found in the indicated promoter regions. The distance in bp relative to the transcription start site (TSS) of every putative κB site is shown, as well as the sequence from the κB site chosen for analysis by ChIP. Chromatin immunoprecipitation analysis of p65 **(B) **or p65acetylated at K314 **(C) **from *p65*(+/+) MEFs kept unstimulated or treated with TNFα for 20 or 180 minutes. Chromatin from *p65*(-/-) MEFs stimulated with TNFα for 180 minutes was used as negative control. Recruitment to the indicated promoters was analyzed. Occupancy to *IP-10 *and *Glucagon *promoters was assessed as positive and negative control, respectively. Samples were normalized to input chromatin and expressed as % input. The result is representative of three independent experiments. Mean values ± SD of three independent runs are shown.

From the above selected genes only *Cfb *was already described to contain a κB site in its promoter [19]. We therefore searched for putative κB sites within the DNA sequence 1 kB upstream of the transcription start site (TSS) of the selected genes. Bioinformatic analysis identified several putative κB sites in the promoters of *Mmp10 *and *Cfb*, one site in the promoter of *Mpa2l*, but none for *Mmp13 *(Fig. 4A). *Mmp13 *was thus not further investigated. ChIP studies in *p65*(+/+) MEFs stimulated with TNFα for 20 and 180 minutes revealed that p65 was recruited to the promoters of *Cfb *and *Mpa2l *in a stimulus-dependent manner, while no enrichment was observed in *p65*(-/-) MEFs (Fig. 4B). These recruitments were promoter-specific, since p65 occupancy to promoter of *glucagon*, a negative control, was not induced upon TNFα stimulation. *IP-10*, a known NF-κB target gene with very well characterized κB sites at its promoter, served additionally as a positive control. Unfortunately, no p65 enrichment could be observed to the κB sites of *Mmp10 *(data not shown), indicating that p65 would activate this gene through other κB sites or other transcription factors.

Interestingly, ChIP experiments using the anti-acetyl K314 antibodies showed that chromatin-associated p65 is indeed acetylated at K314 on all analyzed genes (*Cfb*, *Mpa2l *and *IP-10*) (Fig. 4C), although the expression of these genes was not affected by mutating K314/315 (Fig. 2C and 2D). Furthermore, the detected amount of recruited p65, which was acetylated at K314, increased over time (comparing values at 180 min and 20 min). Unfortunately, the antibody raised against acetyl K315 was not able to immunoprecipitate p65 under the tested conditions (data not shown). Together, these experiments identified *Mpa2l *as a novel NF-κB target gene through the recruitment of p65 to its promoter and provide strong evidence that chromatin-bound p65 is indeed acetylated at K314.

## Discussion

We have previously shown that p65 acetylation plays an important role in regulating NF-κB-dependent transcription of a subset of genes. Our current results confirm those earlier findings by identifying new genes differentially regulated in the acetylation-deficient mutants compared to wild type cells. Remarkably, only a few genes identified after 3 hours TNFα stimulation overlapped with the genes identified in our previous study after 45 minutes TNFα stimulation (data not shown). We also observed that TNFα does not induce NF-κB activity in the complemented MEFs as efficiently as in the parental *p65*(+/+) MEFs used for ChIP (data not shown). Therefore, although our complemented MEFs are useful to address the role of p65 acetylation *in vivo*, we might have identified only part of the genes influenced by p65 acetylation with the micro array screen.

*Cfb *was previously suggested to be a direct target of NF-κB by EMSA experiments [19]. Our ChIP assays confirmed that p65 is recruited to *Cfb *promoter in a TNFα-dependent manner *in vivo *and further identified *Mpa2l *as a novel NF-κB target gene. Although *Mmp10 *and *Mmp13 *were differentially expressed in acetylation-deficient mutant cells compared to wild type cells, we failed to detect p65 recruitment to those promoters. One explanation could be that p65 is recruited to the promoter region of these genes with a different kinetics than the one we investigated here. Alternatively, p65 could bind to a regulatory element located far away from the promoter to regulate transcription, as has been shown for several NF-κB target genes [20-22]. NF-κB is known to activate the expression of many transcription factors and their regulators [4]; consequently, a third possibility is that p65 directly induces the expression of a protein that regulates the expression of these genes. Along this line, NF-κB has been shown to directly activate the expression of the transcription factor Elk-1, which in turn induces *Mmp13 *gene expression [23].

Interestingly, the stimulatory effect of mutating K314/315 was lost when additionally mutating K310, suggesting that K310 acetylation is not affected by the lack of K314/315 acetylation, and that the modification of K310 would counteract the effect of K314/315 at the particular tested genes. Furthermore, the effect of mutating K314/315 on gene expression was more obvious after 3 hours of stimulation compared to early time points (e.g. 20 minutes), suggesting that acetylation of these lysines might more likely affect termination of gene expression than its induction itself. Here, we provide for the first time evidence that endogenous, chromatin-bound p65 is indeed acetylated at K314 upon stimulation with TNFα. Unfortunately, acetylation of K315 could not be confirmed due to the lack of a specific acetylation-dependent antibody that recognizes endogenous proteins. Interestingly, we observed acetylation of p65 K314 at promoters of genes whose expression was not affected by mutating K314/315 (e.g. *IP-10*). Thus, it could well be that p65 is acetylated upon recruitment of p300 at promoters of many genes, but that the expression of only some of these genes is affected by p65 acetylation at distinct lysines.

The observation that mutation of K314/315 increases expression of certain genes (e.g. *Mmp10 *and *Mmp13*) suggests that acetylation of K314 and possibly K315 might represses gene expression. A possible molecular mechanism is that acetylated lysines at p65 create docking sites for bromodomain-containing proteins which need to be recruited to promoters to modulate transcription [24]. On the other hand, we can currently not exclude that lysines 314 and/or 315 are modified by another post-translation modification (e.g. methylation, [25]) and that mutating these residues would thus affect gene expression independently of acetylation. However, provided the observed acetylation of chromatin-bound p65 at K314, acetylation is more likely to compete with another lysine modification, possibly resulting in crosstalk between different modifications [25]. Indeed, a recent study reported that these same two lysines (K314/315) can be methylated by Set9 to induce ubiquitinylation of NF-κB and subsequently terminate gene expression [26]. Thus, acetylation at K314 and possibly K315 could prevent methylation-mediated repression of target genes and thus avoid methylation-induced termination of p65-dependent gene expression. A direct evidence for methylated p65 at K314/315 bound to chromatin is however still missing. Whether the same genes are regulated by both post-translational modifications should be further addressed. Alternatively, both post-translational modifications might regulate distinct set of NF-κB-dependent genes and thus not influence each other.

## Conclusions

Together, our results establish acetylation of K314 as an important regulatory modification of p65 and subsequently of NF-κB-dependent gene expression.

## Methods

### Tissue culture

Complemented *p65*(-/-) NIH 3T3 MEFs stably expressing p65 wild type or the acetylation-deficient mutants were generated by lentiviral complementation as previously described in [18]. Briefly, HEK 293T cells were transfected with the packaging plasmid, the envelope plasmid and pTV-myc-RelA/p65 wild type, RelA/p65 K/R mutants or the control pTV vector. *p65*(-/-) MEFs were infected with the viral supernatant and split into selective medium (2.5 μg/ml Blasticidin) after thirty-six hours. Expression of recombinant proteins was confirmed by western blot analysis. Pools of cells were used for further analysis. They, as well as the *p65*(-/-) and (+/+) parental NIH 3T3 MEFs kindly provided by A. Beg [27], were maintained in DMEM supplemented with 10% FCS, 100 units/ml penicillin/streptomycin (Gibco) and non-essential amino acids (Gibco). HEK 293T cells were maintained in DMEM supplemented with 10% FCS and 100 units/ml penicillin/streptomycin.

### Plasmids

Plasmids for the mammalian overexpression of human p65 wild type and mutants K310R, K314/315R and KTR in HEK 293T cells were described elsewhere [18]. Briefly, pph-CMV-Km-RelA/p65 wild type, previously described in [28], was used as template vector for the generation of the p65 mutants by site-directed mutagenesis according to the QuickChange protocol (Stratagene). All introduced mutations were confirmed by sequencing.

### Reagents and antibodies

Human TNFα, Trichostatin A (TSA), Nicotinamide (Nam) and acetyl-Coenzyme A (acetyl Co-A) were purchased from Sigma. Recombinant mouse TNFα was either purchased from Sigma or generated in our laboratory. Sodium fluoride (NaF) and beta-glycerophosphate were obtained from Fluka. The acetyl-specific antibodies for p65 anti-acetyl K314 (ab18727) and anti-acetyl K315 (ab19869) were generated by Abcam. The following antibodies were purchased from Santa Cruz Biotechnologies: anti-p65 (sc-372) and anti-p300 (sc-585). The anti-myc antibodies were either purchased from Roche (11-667-149-001) or purified from hybridoma cells.

### Generation of recombinant proteins

The recombinant proteins were expressed by baculovirus in Sf21 cells using either the FastBac or the BacPAK systems (Clontech). His-tagged proteins were purified over Ni^2+^-beads (ProBond, Invitrogen).

### Micro array

Complemented cell lines p65 wild type or acetylation-deficient mutants were starved overnight before either left untreated or stimulated with 30 ng/ml TNFα for 3 hours. Total RNA from three biological replicates per sample was isolated at different days using the 'Total RNA isolation mini kit' (Agilent Technologies). RNA quality was measured on the 2100 Bioanalyzer (Agilent Technologies). Microarray experiments were performed using 'Whole Mouse Genome (4 × 44 K) Oligo Microarray Kit' (Agilent Technologies) and 'One-color micro array-based gene expression analysis' (Agilent Technologies) following the manufacturer's protocol. Cy3-labeled cRNA was purified with the RNeasy kit (Qiagen). Dye incorporation was assessed with the ND-1000 Spectrophotometer (NanoDrop Technologies). Per sample, 1.65 μg cRNA from each of the three biological replicates was hybridized to independent arrays according to the manufacturer's protocol. Hybridized slides were scanned with the Agilent DNA Microarray scanner and quantified using the Agilent Feature Extraction software. The data analysis was performed using 'Rosetta Resolver^® ^Gene Expression Data Management and Analysis System' (Rosetta Biosoftware). Briefly, data was processed and normalized with default settings. Then, low-signal genes with signal intensities < 0.1 were filtered out. Differential expression between two conditions was assessed based on the average ratio and significance. All genes with expression ratios < 0.556 and >1.8, and a p-value < 0.05 were selected to generate the tables of significantly regulated genes. These sequence data have been submitted to the GEO database http://www.ncbi.nlm.nih.gov/geo under accession number GSE15196.

### Gene expression by quantitative RT-PCR

Complemented MEFs were starved overnight before treatment with 30 ng/ml recombinant TNFα for different time points. Total RNA was isolated from at least two biological samples at different days with the 'Total RNA isolation mini kit' (Agilent Technologies). RNA quantity was assessed with the ND-1000 Spectrophotometer (NanoDrop Technologies). 2 μg RNA was subsequently retro-transcribed using the 'High-capacity cDNA reverse transcription kit' (Applied Biosystems) following the manufacturer's protocol. Real-time PCR was performed using the Rotor-Gene 3000 (Corbett Life Science, now Qiagen) and TaqMan assays from Applied Biosystems for the following genes: *Cfb, Mpa2l, Mmp10, Mmp13, Rps6 *and *CanX*, according to Applied Biosystems' protocol. The last two genes were used as internal controls to normalize for RNA input. RNA from at least two biological replicates per sample was measured and analyzed with REST [29]. Each experiment was run three independent times, the mean value and ± SD was calculated and blotted into graphs. A 2-tails, paired t-Test was performed with the log of the values to additionally calculate the p-values.

### *In vitro *acetylation assay

1 μg of recombinant p65 wild type or the acetylation-deficient mutants were incubated with 500 ng recombinant p300 in HAT buffer (50 mM Tris HCl pH8, 100 mM NaCl, 10% glycerol, 1 mM PMSF, 1 mM DTT, 1 μg/ml bepstatin, 1 μg/ml leupeptin, 1 μg/ml pepstatin and 1 mM sodium butyrate) with or without addition of 150 μM acetyl-CoA. After 1 hour at 30°C, samples were resolved on SDS-PAGE and analyzed by western blot.

### Acetylation assays in cells

HEK 293T cells were transfected with expression plasmids for p300 and either myc-tagged p65 wild type, the acetylation-deficient mutants or an empty vector, using the calcium phosphate precipitation method. After 23 hours, cells were treated with 10 ng/ml human TNFα for 30 minutes. Then, whole cell extracts were prepared and 40 μg protein was analyzed by SDS-PAGE and western blot.

### Immunoprecipitation

Whole cell extracts from the complemented cells untreated or stimulated with 10 ng/ml TNFα for 40 minutes were used to immunoprecipitate p65. 750 μg of extract were incubated with 1.5 μg of antibody for 1 hour at 4°C in Co-IP buffer (20 mM HEPES pH 7.9, 100 mM NaCl, 2.5 mM MgCl_2_, 0.05% NP-40, 1 mM PMSF, 1 μg/ml pepstatin, 1 μg/ml bestatin, 1 μg/ml leupeptin, 1 μM TSA, 5 mM Nam). Protein G sepharose was added and samples were incubated for another 2 h at 4°C. Samples were washed three times 5 minutes in Co-IP buffer containing 100 mM NaCl before being subjected to 10% SDS-PAGE, followed by western blot.

### Chromatin Immunoprecipitation

*p65*(-/-) or (+/+) MEFs were stimulated with 10 ng/ml mouse TNFα for the indicated time points and fixed with 1% formaldehyde (Calbiochem) for 10 minutes. After extensive washing, the plasma membrane was first lysed with lysis buffer 1 (50 mM Tris HCl pH8, 2 mM EDTA pH8, 0.1% NP-40, 10% glycerol, 1 mM PMSF, 0.5 mM DTT, phosphatase and HDAC inhibitors) and then the nuclear membrane with lysis buffer 2 (50 mM Tris HCl pH8, 5 mM EDTA pH8, 1% SDS, 1 mM PMSF, 0.5 mM DTT, phosphatase and HDAC inhibitors). Chromatin fragmentation was achieved with the Bioruptor (Diagenode). Sonified chromatin was diluted with 9 volumes of dilution buffer (50 mM Tris HCl pH8, 5 mM EDTA pH8, 0.5% NP-40, 200 mM NaCl and 1 mM PMSF) and pre-cleared for 1 hour with Protein A Agarose/salmon sperm DNA (Millipore). 1% of input was saved and the remaining chromatin was then incubated overnight with the specific antibodies. After 30 additional minutes of incubation with Protein A Agarose/salmon sperm DNA, the immuno-complexes were extensively washed with washing buffer (20 mM Tris HCl pH8, 2 mM EDTA pH8, 1% NP-40, 0.1% SDS, 500 mM NaCl and 1 mM PMSF) and then with buffer TE (10 mM Tris HCl pH8 and 1 mM EDTA pH8). Chromatin was eluted with 2% SDS in TE buffer and incubated at 65°C for at least 6 hours. DNA was purified with 'QIAquick PCR purification kit' (Qiagen) following the manufacturer's recommendations and measured by real-time PCR using SYBR Green and the Rotor-Gene 3000 (Corbett Life Science, now Qiagen). The following primers were used: Mpa2l_forward (CAGCCCCTTTTATAGTGAGTC), Mpa2l_reverse (TAC AAAATCCGGGAGTATTGC), Cfb_forward (CACCTGTGAAGCAAGTCTCTCTCT), Cfb_reverse (TTTGTGCAGCAAGGACTCTGACCT), IP-10_forward (GCAATGCCCT CGGTTTACAG), IP-10_reverse (GGCTGACTTTGGAGATGACTCA), Glucagon_ forward (GAGTGGGCGAGTGAAATCAT) and Glucagon_reverse (TGAGCTGCGA ACAGGTGTAG) Samples were normalized to input chromatin and expressed as % input. Each experiment was independently repeated at least three times. Mean values ± SD of three independent real-time PCR runs from one independent ChIP are shown.

## List of abbreviations

acetyl Co-AL: acetyl-CoenzymeA; ChIP: chromatin immunoprecipitation; K: lysine; MEFs: mouse embryonic fibroblasts; Nam: Nicotinamide; NF-κB: nuclear factor κB; TNFα: tumor necrosis alpha; TSA: trichostatin A; TSS: transcription start site.

## Authors' contributions

K.M.R. carried out the molecular studies and drafted the manuscript. M.F. performed a part of the experiments. M.O.H. conceived the study and participated in its design and coordination and helped to draft the manuscript. All authors read and approved the final manuscript.

## Supplementary Material

Additional file 1 **Gene expression analysis of *Mmp10*, *Mmp13*, *Cfb *and *Mpa2l *with quantitative RT-PCR**. The mean value of three independent runs relative to wild type unstimulated is shown, as well as ± SD and p-values. P-values were calculated comparing data of each cell line with wild type. Click here for file
