# Supplementary material for: Acetylation of p65 at lysine 314 is important for late NF-κB-dependent gene expression
Source: BMC Genomics. 2010 Jan 11;11:22. doi: 10.1186/1471-2164-11-22 (PMC2823688; doi:10.1186/1471-2164-11-22)
Supplement: Additional file 1 — Gene expression analysis of Mmp10, Mmp13, Cfb and Mpa2l with quantitative RT-PCR. The mean value of three independent runs relative to wild type unstimulated is shown, as well as ± SD and p-values. P-values were calculated comparing data of each cell line with wild type. [file 1471-2164-11-22-S1.DOC]

**Table S1 A – Gene expression analysis of *Mmp10***

|  | **Wt** | | **K314/315R** | | | **KTR** | | | **pTV** | | |
| --- | --- | --- | --- | --- | --- | --- | --- | --- | --- | --- | --- |
|  | **mean** | **std dev** | **mean** | **std dev** | **p value** | **mean** | **std dev** | **p value** | **mean** | **std dev** | **p value** |
| **0’** | 1.00 |  | 1.17 | 0.53 | 0.85 | 0.79 | 0.12 | 0.11 | 0.76 | 0.11 | 0.08 |
| **20’** | 0.74 | 0.17 | 1.12 | 0.44 | 0.12 | 0.83 | 0.24 | 0.44 | 0.53 | 0.24 | 0.16 |
| **45’** | 0.97 | 0.23 | 1.31 | 0.46 | 0.17 | 1.04 | 0.33 | 0.35 | 0.50 | 0.18 | **0.02** |
| **90’** | 1.80 | 0.26 | 2.29 | 0.80 | 0.38 | 1.75 | 0.54 | 0.65 | 0.82 | 0.30 | **0.05** |
| **180’** | 2.50 | 0.60 | 4.33 | 1.54 | **0.03** | 2.37 | 0.82 | 0.38 | 0.95 | 0.43 | **0.03** |
| **360’** | 3.41 | 0.85 | 5.62 | 2.25 | **0.05** | 3.36 | 1.12 | 0.66 | 0.96 | 0.45 | **0.02** |

**Table S1 B – Gene expression analysis of *Mmp13***

|  | **Wt** | | **K314/315R** | | | **KTR** | | | **pTV** | | |
| --- | --- | --- | --- | --- | --- | --- | --- | --- | --- | --- | --- |
|  | **mean** | **std dev** | **mean** | **std dev** | **p value** | **mean** | **std dev** | **p value** | **mean** | **std dev** | **p value** |
| **0’** | 1.00 |  | 0.98 | 0.30 | 0.80 | 0.57 | 0.06 | **0.01** | 0.07 | 0.04 | **0.02** |
| **20’** | 0.96 | 0.09 | 0.93 | 0.22 | 0.69 | 0.61 | 0.10 | **0.02** | 0.03 | 0.02 | **0.02** |
| **45’** | 1.09 | 0.32 | 1.46 | 0.41 | 0.23 | 0.79 | 0.20 | **0.04** | 0.05 | 0.05 | **0.02** |
| **90’** | 1.55 | 0.25 | 2.03 | 0.73 | 0.51 | 1.36 | 0.57 | 0.44 | 0.08 | 0.07 | **0.03** |
| **180’** | 2.09 | 0.34 | 5.03 | 0.72 | **0.01** | 2.20 | 0.86 | 0.94 | 0.08 | 0.07 | **0.02** |
| **360’** | 2.81 | 0.20 | 4.99 | 1.08 | **0.03** | 2.58 | 0.78 | 0.52 | 0.07 | 0.07 | **0.02** |

**Table S1 C – Gene expression analysis of *Cfb***

|  | **Wt** | | **K314/315R** | | | **KTR** | | | **pTV** | | |
| --- | --- | --- | --- | --- | --- | --- | --- | --- | --- | --- | --- |
|  | **mean** | **std dev** | **mean** | **std dev** | **p value** | **mean** | **std dev** | **p value** | **mean** | **std dev** | **p value** |
| **0’** | 1.00 |  | 0.54 | 0.19 | 0.10 | 0.29 | 0.05 | **0.01** | n.d. |  |  |
| **20’** | 0.92 | 0.06 | 0.40 | 0.14 | **0.04** | 0.23 | 0.05 | **0.00** | n.d. |  |  |
| **45’** | 0.89 | 0.15 | 0.46 | 0.16 | **0.05** | 0.24 | 0.50 | **0.01** | n.d. |  |  |
| **90’** | 1.17 | 0.01 | 0.69 | 0.16 | 0.06 | 0.40 | 0.90 | **0.02** | n.d. |  |  |
| **180’** | 2.13 | 0.32 | 2.03 | 0.56 | 0.48 | 0.93 | 0.13 | **0.00** | n.d. |  |  |
| **360’** | 3.38 | 0.63 | 3.16 | 1.00 | 0.43 | 1.60 | 0.47 | **0.01** | n.d. |  |  |

**Table S1 D – Gene expression analysis of *Mpa2l***

|  | **Wt** | | **K314/315R** | | | **KTR** | | | **pTV** | | |
| --- | --- | --- | --- | --- | --- | --- | --- | --- | --- | --- | --- |
|  | **mean** | **Std dev** | **mean** | **std dev** | **p value** | **mean** | **std dev** | **p value** | **mean** | **std dev** | **p value** |
| **0’** | 1.00 |  | 0.90 | 0.23 | 0.49 | 0.38 | 0.02 | **0.00** | 0.01 | 0.00 | **0.00** |
| **20’** | 0.90 | 0.08 | 0.72 | 0.17 | 0.12 | 0.34 | 0.05 | **0.00** | 0.01 | 0.00 | **0.00** |
| **45’** | 1.05 | 0.16 | 0.90 | 0.22 | 0.22 | 0.40 | 0.04 | **0.01** | 0.01 | 0.00 | **0.00** |
| **90’** | 1.89 | 0.13 | 1.61 | 0.32 | 0.34 | 0.76 | 0.12 | **0.02** | 0.02 | 0.00 | **0.00** |
| **180’** | 2.84 | 0.51 | 3.52 | 0.94 | 0.13 | 1.38 | 0.29 | **0.00** | 0.01 | 0.00 | **0.00** |
| **360’** | 2.39 | 0.47 | 2.60 | 0.76 | 0.38 | 1.08 | 0.28 | **0.00** | 0.00 | 0.00 | **0.00** |
